# Estimating relative risks in multicenter studies with a small number of centers — which methods to use? A simulation study

**DOI:** 10.1186/s13063-017-2248-1

**Published:** 2017-11-02

**Authors:** Claudia Pedroza, Van Thi Thanh Truong

**Affiliations:** Center for Clinical Research and Evidence-Based Medicine, McGovern Medical School at The University of Texas Health Science Center at Houston, 6431 Fannin Street, MSB 2.106, Houston, TX 77030 USA

**Keywords:** Multicenter studies, Relative risk, Generalized linear mixed models, Generalized estimating equations, Correlated binary data, Bayesian log binomial

## Abstract

**Background:**

Analyses of multicenter studies often need to account for center clustering to ensure valid inference. For binary outcomes, it is particularly challenging to properly adjust for center when the number of centers or total sample size is small, or when there are few events per center. Our objective was to evaluate the performance of generalized estimating equation (GEE) log-binomial and Poisson models, generalized linear mixed models (GLMMs) assuming binomial and Poisson distributions, and a Bayesian binomial GLMM to account for center effect in these scenarios.

**Methods:**

We conducted a simulation study with few centers (≤30) and 50 or fewer subjects per center, using both a randomized controlled trial and an observational study design to estimate relative risk. We compared the GEE and GLMM models with a log-binomial model without adjustment for clustering in terms of bias, root mean square error (RMSE), and coverage. For the Bayesian GLMM, we used informative neutral priors that are skeptical of large treatment effects that are almost never observed in studies of medical interventions.

**Results:**

All frequentist methods exhibited little bias, and the RMSE was very similar across the models. The binomial GLMM had poor convergence rates, ranging from 27% to 85%, but performed well otherwise. The results show that both GEE models need to use small sample corrections for robust SEs to achieve proper coverage of 95% CIs. The Bayesian GLMM had similar convergence rates but resulted in slightly more biased estimates for the smallest sample sizes. However, it had the smallest RMSE and good coverage across all scenarios. These results were very similar for both study designs.

**Conclusions:**

For the analyses of multicenter studies with a binary outcome and few centers, we recommend adjustment for center with either a GEE log-binomial or Poisson model with appropriate small sample corrections or a Bayesian binomial GLMM with informative priors.

**Electronic supplementary material:**

The online version of this article (doi:10.1186/s13063-017-2248-1) contains supplementary material, which is available to authorized users.

## Background

In multicenter studies, outcomes from the same center cannot be assumed to be independent, and analyses often need to account for center clustering. Neglecting to account for center may lead to erroneous conclusions, particularly when randomization is stratified by center [1–4]. Yet, authors of a recent review of multicenter studies published in four major medical journals (*BMJ*, *New England Journal of Medicine*, *JAMA*, and *The Lancet*) found that only 22% of randomized controlled trials (RCTs) with a binary outcome reported accounting for a center effect, a rate similar to past reviews [5, 6]. This result may be due to the fact that it is challenging to properly adjust for center when there are few centers, total sample size is small, or there are few events per center. Clear practical guidelines for the statistical analyses and reporting of multicenter studies are needed to assist investigators and data analysts in conducting appropriate multicenter analyses.

The best-suited methods to adjust for center include generalized estimating equations (GEEs) and generalized linear mixed models (GLMMs; also referred to as *random effects*, *multilevel*, or *mixed effects models*). However, careful application of these methods is needed for studies with few centers. For example, the robust SEs typically reported for GEEs are downward-biased when the number of centers is < 50 [7]. For GLMMs, the approximate Wald test and CIs may have inflated type I error rates [8].

Our objective in this article is to review and evaluate both frequentist and Bayesian state-of-the-art statistical methods for estimating relative risk (RR) for multicenter studies. We focus on RRs rather than ORs because RRs are considered a more meaningful and interpretable treatment measure [9, 10], and few studies have evaluated methods for estimating adjusted RRs. We provide recommendations and practical guidelines for analyzing both RCTs and observational multicenter studies.

## Methods

We review three methods for estimating RRs: log-binomial regression, the widely used GEE method, and GLMMs. Specifically, we evaluate a total of ten regression models:

1. Log binomial
2. GEE binomial
3. GEE binomial with small-sample correction of SEs
4. GEE Poisson
5. GEE Poisson with small-sample correction of SEs
6. GLMM binomial
7. GLMM binomial with bootstrapped SEs
8. GLMM Poisson
9. GLMM Poisson with bootstrapped SEs
10. Bayesian GLMM binomial

We assume a study design with ≤ 30 centers, binary outcome, binary treatment/exposure variable, and a binary baseline covariate that could be a stratifying variable or a potential confounder. We also assume the center size variation is not very large (i.e., coefficient of variation < 0.40). Table 1 provides a summary of all the models and details of their specification evaluated in the simulation study.

**Table 1:** Summary of details of ten regression models evaluated in the simulation study

Model	SEs	95% CI or posterior interval	Other assumptions
GLM-Bin	Model-based, unadjusted for center correlation	Wald	
GEE binomial	Robust sandwich	*t*-based	Exchangeable working correlation
GEE binomial KC-corrected^a^	Robust sandwich with small-sample correction	*t*-based	Exchangeable working correlation
GEE Poisson	Robust sandwich	*t*-based	Exchangeable working correlation
GEE Poisson KC-corrected^a^	Robust sandwich with small-sample correction	*t*-based	Exchangeable working correlation
GLMM binomial	Model-based	Wald	Adaptive quadrature with 10 points
GLMM binomial bootstrap^a^	Parametric bootstrap	Parametric bootstrap, quantile-based	Laplace for fitting bootstrap samples
GLMM Poisson	Model-based	Wald	Adaptive quadrature with 10 points
GLMM Poisson bootstrap^a^	Parametric bootstrap	Parametric bootstrap, quantile-based	Laplace for fitting bootstrap samples
Bayesian binomial GLMM	Posterior SD	Quantile-based posterior interval	Priors β_0_ ~ Normal(0,10^2^); β_1_, β_2_ ~ Normal(0,1); σ ~ half-Normal(0,1)

*Abbreviations: GEE* Generalized estimating equation, *GLM-Bin* Log-binomial regression model, *GLMM* Generalized linear mixed model, *KC* Kauermann and Carroll

^a^The small sample KC correction or bootstrap samples correct only the SEs and 95% CIs and do not affect the point estimates of the risk ratio

We do not investigate methods treating center as a fixed effect, because problems with this approach (including exclusion of patients or centers, biased treatment effect estimate, and increased type I error) have been noted before [2–4].

### Log-binomial regression

Binary regression models (i.e., logistic) without any adjustment for center correlation are the most often used methods for analyzing multicenter studies. To estimate RRs instead of ORs, we can use a log-binomial regression model (GLM-Bin) adjusting for covariates but with no adjustment for center correlation. Letting *y*_*ij*_ be the observed binary outcome (yes/no) for subject *i* in center *j*, this model is specified as

1 $$ \mathit{\log}\left({\mathrm{p}}_{\mathrm{ij}}\right)={\beta}_0+{\beta}_1{\mathrm{x}}_{1\mathrm{ij}}+{\beta}_2{\mathrm{x}}_{2\mathrm{ij}}, $$

where *p*_*ij*_ is the probability of the outcome *y*_*ij*_, *x*_1*ij*_ is the treatment/exposure indicator, and *x*_2*ij*_ is a binary baseline covariate. A Bernoulli distribution is assumed for *y*_*ij*_. The RR of the treatment/exposure is given by exp(β_1_). Model-based SEs (obtained from the regression model and unadjusted for center clustering) and Wald-type 95% CIs [$\widehat{\upbeta}$_1_ ± 1.96 × SE($\widehat{\upbeta}$_1_)] are usually reported for this model. In addition to ignoring within-center correlation, this model also has the disadvantage of convergence problems at the parameter boundary and can lead to probability estimates > 1.

### GEE models

The GEE log-binomial and log Poisson models take the same form as model (1), assuming either a binomial or Poisson distribution for *y*. However, the SEs are corrected by using GEEs with an exchangeable correlation matrix, which assumes that patient outcomes from the same center are correlated but are independent from patient outcomes in different centers. GEE Poisson (also referred to as *modified Poisson*) regression is widely used to estimate RRs because it provides consistent estimates of the RR and is more stable than the GEE binomial model [9, 11]. Similarly to the GLM-Bin, probability estimates from both of these GEE models can be > 1.

Robust sandwich SEs for possible misspecification of the covariance structure (and misspecification of the distribution for Poisson regression) are typically used with GEE methods [12]. When the number of centers is small, a bias-corrected variance sandwich estimator is needed to provide correct inference [13]. We use the Kauermann and Carroll (KC) [14] correction of robust SEs because it has been shown to perform well with small numbers of centers [15].

Similarly, the Wald test and CIs typically reported for GEEs have been noted to have inflated type I errors with few centers [15, 16]. An approximate *t* statistic that accounts for the large variation in the sandwich estimator often present with small samples has been shown to perform better than the Wald test in this setting [17].

For both the binomial and Poisson GEE models, we assess the performance of (1) robust SEs coupled with approximate *t*-based 95% CIs [$\widehat{\upbeta}$_1_ ± *t*_*d*_ × SE_robust_($\widehat{\upbeta}$_1_)] and (2) KC-corrected robust SEs with *t*-based 95% CIs for the RR.

### GLMMs

Random effects models account for within-center correlation by including a random center intercept. Estimates derived from GLMMs are interpreted as center-specific (or conditional) as opposed to the marginal interpretation of GEE estimates. However, we note that under a log link, the estimated treatment/exposure effect is the same for GEE and GLMMs [18]. Again letting *y*_*ij*_ be the observed binary outcome for subject *i* in center *j*, a binomial GLMM is specified as

2 $$ {\displaystyle \begin{array}{c}{\mathrm{y}}_{\mathrm{ij}}\sim \mathrm{Bernoulli}\left({\mathrm{p}}_{\mathrm{ij}}\right)\\ {}\mathit{\log}\left({\mathrm{p}}_{\mathrm{ij}}\right)={\upbeta}_0+{\upbeta}_1{\mathrm{x}}_{1\mathrm{ij}}+{\upbeta}_2{\mathrm{x}}_{2\mathrm{ij}}+{\mathrm{u}}_{\mathrm{j}}\\ {}{\mathrm{u}}_{\mathrm{j}}\sim \mathrm{Normal}\left(0,{\upsigma}^2\right),\end{array}} $$

where *u*_*j*_ is the random center effect and *x*_1*ij*_ and *x*_2*ij*_ are the treatment/exposure indicator and baseline binary covariate, respectively.

We fit GLMMs using adaptive Gauss-Hermite quadrature with 10 quadrature points to provide a good compromise between accuracy and computation time in the simulation study [19]. We use two methods to compute SEs and 95% CIs. First, we calculate Wald 95% CIs for the RR. Although *t* statistics with various methods for calculating the degrees of freedom have been proposed as a better alternative to the Wald test, these are still approximations in GLMMs and may not perform well, particularly with few centers [8]. Hence, in the second method, we use a parametric bootstrap to calculate SEs and 95% CIs, which is a better alternative for computing CIs for GLMMs [20].

We also assess the performance of log Poisson GLMM using model-based SEs coupled with Wald 95% CIs and compare them with those obtained from a parametric bootstrap. We again note that probability estimates from these GLMMs may be > 1.

### Bayesian binomial GLMM

A Bayesian approach provides several advantages, including the ability to give direct estimates of probability of benefit or harm from an intervention or exposure [21]. For the binomial GLMM, weakly informative prior distributions help stabilize the parametric estimates and hence address the convergence issues often seen with the frequentist approach [10, 22–24]. Constraints on the parameters are also easily implemented to avoid probability estimates > 1 [22]. In contrast to frequentist methods, Bayesian SEs and credible intervals (CrIs) for the RR account for all uncertainty in the model, including the between-center variation. Another advantage is that Bayesian inference does not rely on asymptotic results, which is an important issue when the number of centers is limited. A Bayesian approach also allows for the inclusion of informative priors derived from external information to exclude unrealistic RR values [25, 26].

We investigate the performance of a Bayesian GLMM with the same form as that in model 2. For prior distributions, we use neutral priors for all parameters to represent equipoise: a Normal(0,10^2^) for the intercept β_0_, Normal(0,1) for β_1_ and β_2_, and half-Normal(0,1) for σ. We use slightly informative priors on β_1_ and β_2_ with a 95% CrI of 0.14–7 in the RR scale to exclude unrealistic RR values that are almost never observed in studies of medical interventions. Similar priors skeptical of large treatment effects have been studied and shown to have good operating characteristics even with small sample sizes [26]. The half-Normal prior for the SD of the random center effect σ is a weakly informative prior that has been shown to perform well [27]. We constrain all *p*_*ij*_ < 1 in the model (*see* Additional file 1 for sample code).

### Simulation study

We conducted a simulation study assuming both a multicenter two-arm RCT and a multicenter observational study design. For each scenario, we simulated 1000 datasets from model 2 with 4, 10, or 30 centers. The number of subjects per center was sampled from a Poisson distribution with mean of 10, 20, and 50 to give average (expected) total samples sizes ranging from 40 to 1500. Under the RCT scenarios, randomization was stratified by center using permuted blocks of size 4. The covariate *x*_2*ij*_ was generated from Bernoulli(0.3), and the random center effect u_j_ from Normal(0,0.4) to induce an intracluster correlation coefficient (ICC) of 0.08, where σ^2^ = ICC×(1−$\overline{\mathrm{p}}$)/$\overline{\mathrm{p}}$ and $\overline{\mathrm{p}}$ is the average probability in the sample [28]. The ICC represents the degree of dependence or correlation among observations from individuals within the same cluster or center [27]. The ICC value used in this simulation is within the range of values previously reported in cluster clinical trials [29].

For all RCT scenarios, we assumed a control outcome rate of 15% [i.e., exp(β_0_) = 0.15]. The treatment and covariate effects were both set to an RR of 1.5 [i.e., β_1_ = β_2_ = log(1.5)]. Whenever the simulated *p*_*ij*_ was > 1, a new value of the random center effect *u*_*j*_ was sampled until *p*_*ij*_ < 1.

For the observational study scenarios, we assigned half of the subjects to exposure and the other half to a nonexposure group. To induce confounding, we generated the binary covariate *x*_2*ij*_ with prevalence of 0.4 in the exposure group and 0.2 in the nonexposed group using a discretized multivariate Normal method [30]. All other methods and parameters were the same as under the RCT scenarios.

Each dataset was analyzed using all the methods listed in Table 1. For the binomial and Poisson GLMMs, we used 3000 bootstrap samples for each dataset to calculate the bootstrap SEs and 95% CIs (from the quantiles). To speed up the calculation, we used Laplace approximation when fitting the models to the bootstrap samples.

The Bayesian GLMM was fitted via Markov chain Monte Carlo (MCMC) methods [31]. We used 3 MCMC chains, each with 10,000 iterations using the first 2000 as burn-in. Starting values were sampled from the estimated coefficients and SEs of the frequentist log Poisson model. We visually inspected the trace plots of all estimated parameters for the first 50 datasets of each scenario to monitor convergence of the chains. We additionally calculated the convergence diagnostic $\widehat{\mathrm{R}}$ and deemed any datasets with an $\widehat{\mathrm{R}}$ > 1.1 for any parameter as exhibiting nonconvergence (*see below*) [32]. We captured the posterior median of all four parameters and the 2.5% and 97.5% percentiles of β_1_ to calculate coverage of the 95% posterior interval. As a sensitivity analysis, for scenarios with ten subjects per center, we also fitted the Bayesian GLMM using vague Normal(0,10^4^) priors for β_0_, β_1_, and β_2_ and half-Cauchy(0,1) [33] for σ.

For all models, we calculated the bias (β_estimate_ − β_true_), root mean square error (RMSE), coverage of the 95% CI or posterior interval for the treatment/exposure effect β_1_, and convergence rate. We defined convergence as the percentage of simulated datasets where (1) the model converged (i.e., no error messages); (2) the absolute values of the point estimates for β_0_, β_1_, and β_2_ were < 5 (larger values would indicate unstable estimates); and (3) for the Bayesian models, the $\widehat{\mathrm{R}}$ values for all parameters were < 1.10. For each model and scenario, we assessed bias, RMSE, and coverage only in datasets where convergence was achieved.

All simulations and analyses were conducted in R [34]. For the fitting of GEE models, we used the geepack [35] and geesmv [36] packages to calculate the degrees of freedom for the *t* statistic and the KC-corrected robust SEs. For GLMMs, we used the lme4 package [37] for the frequentist models and Stan [38] through the R interface rstan [39] to fit the Bayesian models. We provide sample code in Additional file 1.

## Results

### Convergence

For the Bayesian models, trace plots of the parameters showed the three chains mixing well after burn-in, except for a small percentage of the datasets, where one of the MCMC chains of σ failed to converge near 0 for a portion of the chain. (Other parameters also did not converge; an example of an RCT dataset is shown in Additional file 1: Figure S1.) These convergence issues were also detected by the $\widehat{\mathrm{R}}$ diagnostic (>1.1), and these datasets were excluded from the results. Convergence rates for Bayesian models ranged from 92% for the RCT scenario with 4 centers and 10 subjects per center to 100% for some scenarios with 10 or 30 centers. Convergence rates for all scenarios are shown in Additional file 1: Table S1.

All frequentist models exhibit convergence problems for the smallest sample size for both designs, with convergence rates ranging from 45% for the binomial GLMM to 86% for the GEE Poisson model. For all other scenarios, convergence was not an issue, except for the binomial GLMM, which had poor convergence rates for all scenarios. Its lowest convergence rate was 27% for the scenario with 30 centers with 10 subjects/center (Additional file 1: Table S1).

### Bias and RMSE

**Fig. 1:**
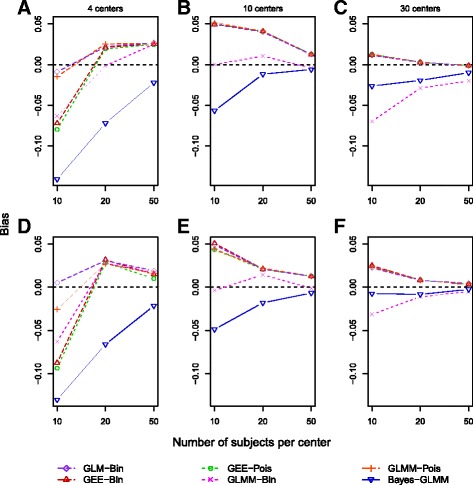
Bias of the estimates of β_1_ (calculated as β_estimate_ − β_true_) for different scenarios under a multicenter randomized controlled trial study design (**a**–**c**) and an observational study design (**d**–**f**) based on 1000 simulations for each scenario. All scenarios used a β_1_ of log(1.5), a control outcome rate of 15%, and an intracluster correlation coefficient of 0.08. *GEE* Generalized estimating equation, *GLM-Bin* Log-binomial regression model, *GLMM* Generalized linear mixed model

**Fig. 2:**
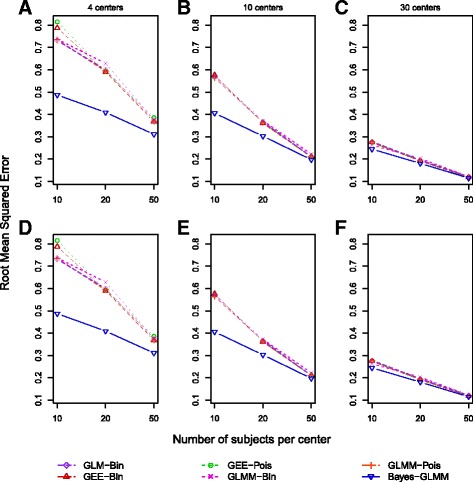
Root mean square error of β_1_ for scenarios under randomized controlled trial study designs (**a**–**c**) and observational study designs (**d**–**f**) based on 1000 simulations for each scenario. All scenarios used a β_1_ of log(1.5), a control outcome rate of 15%, and an intracluster correlation coefficient of 0.08. *GEE* Generalized estimating equation, *GLM-Bin* Log-binomial regression model, *GLMM* Generalized linear mixed model

The bias was generally small for all frequentist models. It was larger for the smallest sample sizes and diminished as the number of centers and total sample size increased. The Bayesian estimates were more biased in the smallest sample sizes. The negative bias indicates that the posterior medians of the treatment effect are shrunk toward 0 because of the influence of the informative priors. The effect of the prior on the posterior estimates and the resulting bias from the Bayesian GLMM diminishes as the sample size increases and is smaller than the bias from frequentist models for some scenarios (Fig. 1). The Bayesian models have the smallest RMSE for scenarios with four or ten centers. All models give very similar RMSEs with 30 centers (Fig. 2). The bias and RMSE were very similar for both study designs.

### Coverage

**Fig. 3:**
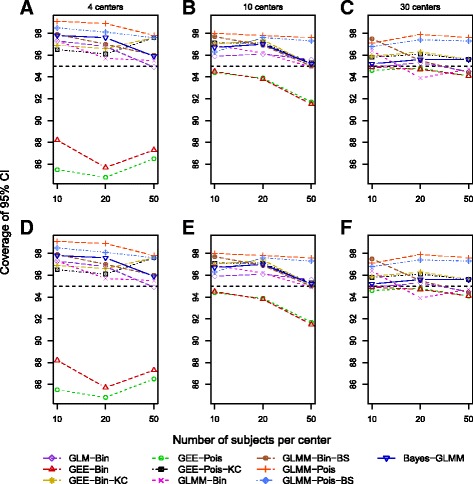
Coverage of 95% CI and posterior interval of β_1_ for scenarios under randomized controlled trial study designs (**a**–**c**) and observational study designs (**d**–**f**) based on 1000 simulations for each scenario. All scenarios used a β_1_ of log(1.5), control outcome rate of 15%, and an intracluster correlation coefficient of 0.08. *GEE* Generalized estimating equation, *GLM-Bin* Log-binomial regression model, *GLMM* Generalized linear mixed model, *KC* Kauermann and Carroll, *BS* Bootstrap

Figure 3 shows coverage of the 95% CIs and posterior interval for β_1_. The unadjusted GLM-Bin exhibits coverage above the nominal range (93.6–96.4% using 1000 datasets) with 4 or 10 centers and 10 or 20 subjects/center, but it has good coverage for all other scenarios. GEE CIs without the KC correction also have poor coverage with 4 centers (85–87%) and 10 centers with 50 subjects (~92%). KC-corrected GEE CIs have good coverage across the majority of scenarios, with the GEE Poisson having coverage closer to nominal than the GEE binomial. The binomial GLMM has coverage above the nominal value for scenarios with the three smallest total sample sizes; the coverage of the bootstrap CIs is more conservative than the Wald CIs for some scenarios. However, the GLMM Poisson model results in coverage well above the nominal value in all scenarios with both Wald-type and bootstrap CIs. The Bayesian model has conservative coverage for scenarios with total sample size ≤ 200; for all other scenarios, coverage is close to the nominal value. The study design had little effect on coverage.

### Sensitivity results

Under both study designs with four centers, Bayesian GLMMs with vague priors had a lower convergence rate, smaller bias, larger RMSE, and less conservative coverage than informative priors. For scenarios with 10 or 30 centers, vague priors resulted in similar convergence rate, bias, RMSE, and coverage compared with informative priors for both designs. In all scenarios, the estimates of the between-center SD were very similar under both vague and informative priors.

## Examples

### Infection treatment multicenter trial

We analyzed the data presented by Beitler and Landis [40] arising from an eight-center RCT investigating the efficacy of an active drug compared with control for treatment of an infection. The primary outcome was favorable response to the drug. In the eight centers, the rate of success in the active drug group (*n* = 130) varied from 9% to 80%, whereas the control group (n = 143) had a rate of success ranging from 0 to 86%. We used the same methods as described for the simulation study (excluding Poisson GLMM, which did not perform well in the simulation study). Table 2 shows the estimated RRs derived from the different methods. The binomial GLMM model did not converge. The RR estimates differ across the models, with the binomial GEE resulting in the largest RR of 1.43 and the Bayesian GLMM resulting in the smallest RR of 1.27. The 95% CIs from the GEE models without the KC correction do not include 1.0. In comparison, the 95% CI from the unadjusted GLM and KC-corrected GEE models include 1.0. The Bayesian 95% CrI is the narrowest, despite properly accounting for center variation (estimated as 0.81) and might lead to a conclusion that the active drug is effective.

**Table 2:** Estimated relative risk and 95% CI or credible interval for multicenter randomized controlled trial example

Model^a^	RR	95% CI or CrI
GLM-Bin	1.29	0.95–1.75
GEE binomial	1.43	1.01–2.02
GEE binomial, KC-corrected		0.96–2.14
GEE Poisson	1.42	1.01–2.01
GEE Poisson, KC-corrected		0.95–2.12
GLMM binomial^b^	–	–
Bayesian binomial GLMM	1.27	1.00–1.65

*Abbreviations: CrI* Credible interval, *GEE* Generalized estimating equation, *GLM-Bin* Log-binomial regression model, *GLMM* Generalized linear mixed model, *KC* Kauermann and Carroll, *RR* Relative risk

Except for the GLM-Bin model, all models are adjusted for center correlation. The KC bias correction in the GEE models adjusts the robust SE for the small number of centers (estimate of RR does not change)

^a^GLMM Poisson models are excluded because of their poor performance in the simulation study

^b^Model did not converge

### Pediatric appendicitis observational study

In this study, we compared cohorts of children before and after implementation of a clinical practice guideline for treatment of perforated appendicitis in children [41]. The study was conducted in a children’s hospital with 11 surgeons providing care. The primary outcome was the occurrence of any adverse event, such as readmission or surgical site infection. Totals of 191 and 122 patients were included in the pre- and postimplementation cohorts, respectively. We compared the analytical methods including identification of intra-abdominal abscess as a covariate, with surgeon as the clustering variable. Estimated RRs and 95% CIs are shown in Table 3. Compared with the other models, the RR estimates from both GEE models are closer to 1 and their CIs without the KC bias correction are narrower than all other intervals. However, the main conclusion of the intervention being associated with reduced adverse outcomes (although not statistically significant) would not differ on the basis of the analytical method chosen.

**Table 3:** Results for pediatric appendicitis study

Model	RR	95% CI or CrI
GLM-Bin	0.76	0.51–1.11
GEE binomial	0.79	0.56–1.13
GEE binomial, KC-corrected		0.53–1.19
GEE Poisson	0.80	0.56–1.14
GEE Poisson, KC-corrected		0.53–1.21
GLMM binomial	0.76	0.51–1.11
GLMM binomial bootstrap		0.51–1.10
Bayesian binomial GLMM	0.73	0.49–1.07

*Abbreviations: CrI* Credible interval, *GEE* Generalized estimating equation, *GLM-Bin* Log-binomial regression model, *GLMM* Generalized linear mixed model, *KC* Kauermann and Carroll, *RR* Relative risk

The KC bias correction in the GEE models adjusts the robust SE for the small number of centers (estimate of RR does not change)

## Discussion

For multicenter studies, it is important to adjust for possible center correlation when computing treatment effects, particularly when the proportion of center variability over total variability is large or when randomization is stratified by center to ensure correct SEs and CIs [1, 4]. However, no clear guidelines exist for the appropriate analyses, and it can be challenging for data analysts to perform a properly adjusted analysis when the outcome is binary and there are few centers. In this paper, we have reviewed and evaluated methods available for analyses of multicenter studies.

### Summary of simulation results

For all but the smallest sample size, convergence rates were ≥ 96% for all models except the binomial GLMM. This model had convergence problems in all scenarios investigated, and its use may be limited. All frequentist estimates of the treatment effect had small and very similar bias. The Bayesian estimates were more biased in the smallest sample sizes. Although the binomial GLM with unadjusted SEs had very little bias, it had conservative coverage for the smallest sample sizes in both the RCT and observational designs.

The GEE models without a small sample correction for the sandwich SEs had very poor coverage with four or ten centers, even when coupled with approximate *t*-based CIs. This poor performance of sandwich SEs has been noted before in estimating ORs [3]. Using the KC bias correction greatly improved the performance of these models across all scenarios. These results are similar to those obtained by Yelland et al. [9] and Zou and Donner [11], although they used different corrections for the variance estimates.

When the binomial GLMM achieved convergence, it had good overall performance except for sample sizes < 100, where it had conservative coverage. Across all scenarios, the Poisson GLMM also had coverage above the nominal value even with bootstrapped CIs, which would lead to diminished power.

The Bayesian GLMM had good coverage across all scenarios, and the bias exhibited in the smallest sample sizes was only slightly larger than the other models evaluated. Its higher convergence rate for the smallest sample size is due to the use of informative priors that help stabilize the estimates, particularly in cases of complete separation [24, 26].

Although we do not report the estimate of the SD of the random effects (or ICC), the Bayesian GLMM outperformed the frequentist models with estimates that were very close to the true parameter value. In contrast, the frequentist models consistently underestimated this parameter even in the larger sample sizes. This downward bias of estimates of the variance components in GLMMs is well known [19, 42]. Here the Bayesian approach has a clear advantage over frequentist methods because it provides less biased variance estimates and automatically produces CIs for these parameters. More importantly, the point estimates and CIs of the treatment/exposure effect appropriately account for the uncertainty in the variance parameter.

The sensitivity analysis for the Bayesian GLMM using vague priors produced results very similar to those under the informative priors. However, the range of effect sizes supported by the vague priors is unrealistic, and we strongly recommend against using these priors.

### Recommendations

On the basis of our simulation results and other studies [3, 43], we recommend that both RCT and observational multicenter studies adjust for center in the analysis. Although adjusting for center when the ICC is small may not provide a great advantage, it also does not adversely affect the point estimates, SEs, and type I error rates. Furthermore, methods that properly adjust for center clustering are easy to implement in most statistical software.

We do not recommend the use of a Poisson GLMM to estimate adjusted RR. We do recommend use of a binomial GLMM, except when the number of centers is < 5 or the total sample size is < 100, although convergence may be a problem. The most robust frequentist methods appear to be either a GEE log-binomial model or a Poisson model with an exchangeable correlation. When the number of centers or clusters is < 50, a sandwich variance estimator needs to include a small sample correction such as the KC correction used here. Kahan et al. [3] reported that model-based SEs may be another option for GEE models, but we did not evaluate them here.

A Bayesian GLMM is a robust alternative. This method performed the best in terms of all measures of convergence, bias, RMSE, and coverage. Another advantage of a Bayesian approach is that exterior information about treatment/exposure effect can be formally incorporated into the prior distributions. As we did here, the priors can explicitly exclude large effects, which are unlikely in clinical studies. Probabilities of benefit or harm are easily obtained and can be more informative for investigators than the traditional *p* value. Although our focus was not on the estimation of the between-center variance, the Bayesian model outperformed all other methods in estimating this parameter. Implementation of the Bayesian model can be done in OpenBUGS or in Stan through R as was done here.

### Limitations

Our simulation study was limited to one treatment/exposure effect size and control rate. However, others have noted similar performance when the effect size or control outcome rate was varied [3, 9, 11, 15]. We also did not investigate fixed effects models, because their limitations have been noted before [2–4]. However, these methods could be an alternative method of analysis for studies with three or fewer centers. We also note computational limitations faced in most simulation studies. In particular, results from the Bayesian models would have benefited from running longer chains. Increasing the number of bootstrap samples for the GLMMs could also potentially improve their performance. Our simulation study did not include scenarios with an ICC of 0. However, others have found that the methods recommended here perform well even in cases where the ICC is very close to 0 [8, 15, 43]. We investigated the performance of only an exchangeable correlation matrix for the GEE models, which is a plausible assumption for multicenter data. However, other correlation structures can be used, and the performance of GEE models has been shown to be robust to the choice of the correlation structure [11].

We chose to focus on binary outcome because it is the most common type of outcome reported in medical research, but it would be important to investigate methods for other types of outcomes (i.e., time-to-event data). Last, we did not investigate treatment by center interactions, and this is an important issue that needs to be investigated in future studies.

## Conclusions

For the analysis of multicenter studies with a binary outcome, we recommend adjustment for center with either a GEE log-binomial or Poisson model or a Bayesian binomial GLMM with informative priors. The GEE models should include a small sample variance correction for sandwich estimators when the number of center is < 30. The Bayesian model with informative priors provides stable estimates, greater flexibility, and good performance even with very small sample sizes.

## Additional file

Additional file 1:
**Figure S1.** Trace plots of parameters for an example dataset exhibiting convergence issues. **Table S1.** Convergence rates for regression models based on 1000 simulated datasets. Sample R code for fitting models used in the simulation study. (PDF 431 kb)

## Abbreviations

CrI: Credible interval
GEE: Generalized estimating equation
GLM-Bin: Log-binomial regression model
GLMM: Generalized linear mixed model
ICC: Intracluster correlation coefficient
KC: Kauermann and Carroll
MCMC: Markov chain Monte Carlo
RCT: Randomized controlled trial
RMSE: Root mean squared error
RR: Relative risk
