# Supplementary material for: Estimating relative risks in multicenter studies with a small number of centers — which methods to use? A simulation study
Source: Trials. 2017 Nov 2;18:512. doi: 10.1186/s13063-017-2248-1 (PMC5667460; doi:10.1186/s13063-017-2248-1)
Supplement: Additional file 1: — Figure S1. Trace plots of parameters for an example dataset exhibiting convergence issues. Table S1. Convergence rates for regression models based on 1000 simulated datasets. Sample R code for fitting models used in the simulation study. (PDF 431 kb) [file 13063_2017_2248_MOESM1_ESM.pdf]

Supplementary Material

Supplement to: Estimating relative risks in multicenter studies with small number of centers — which methods to use?

Claudia Pedroza and Van Thi Thanh Truong

1 Simulation Results

eFigure 1: Trace plots for an example dataset exhibiting convergence issues. It is from the scenario with 4 centers and 10 subjects per center under the randomized controlled trial study design. We note that the MCMC chain 3 of ‘sigma’ gets stuck at 0 causing the other parameters to also get stuck.

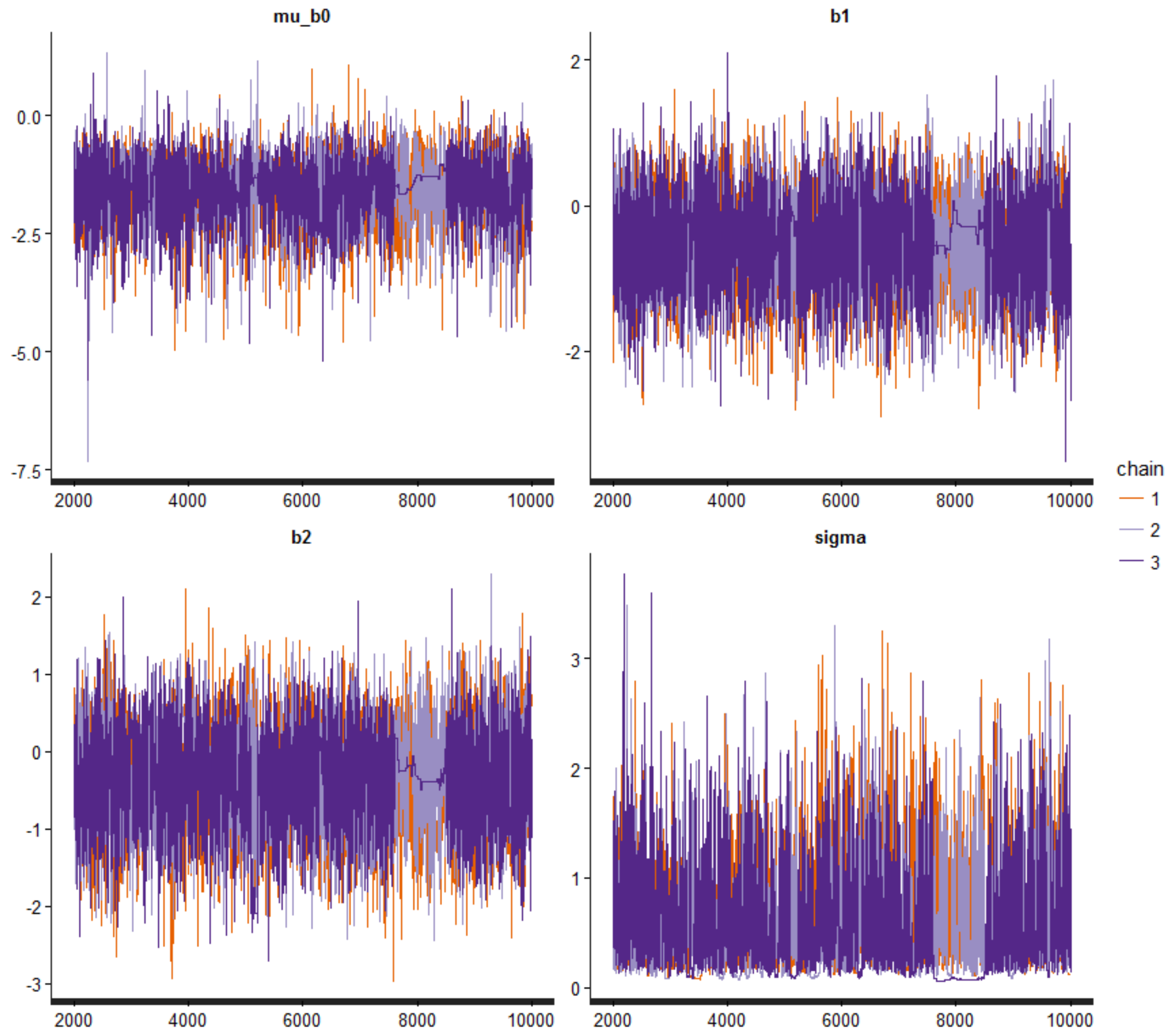

eTable 1. Convergence rates (%) for 6 models based on 1000 simulated datasets for a multicenter RCT design and multicenter observational design (in parentheses). The Bayesian model with vague priors was only fitted to datasets from scenarios with 10 subjects per center.

Number of centers	Number of subjects per center	GLM log binomial	GEE log binomial	GEE log Poisson	GLMM log binomial	GLMM log Poisson	Bayesian binomial GLMM	Bayesian binomial GLMM with vague priors
4	10	78 (75)	82 (80)	86 (83)	47 (45)	82 (81)	92 (97)	76 (77)
	20	96 (97)	96 (96)	97 (97)	69 (70)	97 (97)	98 (98)	
	50	100 (100)	100 (100)	100 (100)	84 (85)	100 (100)	99 (99)	
10	10	100 (99)	99 (100)	99 (100)	48 (51)	99 (100)	97 (98)	97 (96)
	20	100 (100)	100 (100)	100 (100)	59 (61)	100 (100)	98 (99)	
	50	100 (100)	100 (100)	100 (100)	68 (67)	100 (100)	100 (99)	
30	10	100 (100)	100 (100)	100 (100)	33 (27)	100 (100)	98 (99)	98 (98)
	20	100 (100)	100 (100)	100 (100)	31 (31)	100 (100)	99 (100)	
	50	100 (100)	100 (100)	100 (100)	34 (32)	100 (100)	100 (100)	

2 Sample R and Stan code for fitting models evaluated in simulation study

Sample code is given below to fit the models presented in the paper. We assume that the data consists of the binary outcome variable 'y', treatment/exposure group variable 'x1', baseline predictor 'x2', and cluster variable 'center.' Input for the functions is the dataset (sorted by center id). Two functions, '`GEE.var.lz.BIN`' and '`GEE.var.kc.BIN`' needed to calculate degrees of freedom for the GEE log binomial model are given at the end of the code. These functions are modifications of the '`GEE.var.lz`' and '`GEE.var.kc`' functions in the `geesmv` package.

For the Bayesian binomial GLMM, the model assumes mildly informative priors as described in Table 1 of the paper but can be easily modified. We additionally provide OpenBUGS code to implement the same model. Note that the Normal distributions in OpenBUGS are parameterized using precision, which is 1/variance.

R and Stan code

```
library(rstan)
library(truncnorm)
library(geepack)
library(geesmv)
library(gee)
library(matrixcalc)
library(lme4)

df2<-read.csv("C:/Users/cpedroza/Google
Drive/MulticenterTrialsAnalysis/Code/CohortSurg.csv",head=T)

names(df2)<-c("y","x1","x2","center")

df2<-df2[order(df2$center),]

#------------------------------------------------------------
#-- BINOMIAL GLM WITHOUT ADJUSTMENT FOR CENTER CORRELATION -----
m1<-glm(y~x1+x2,family=binomial(link="log"),data=df2)

#RR and 95% Wald CI
exp(coef(m1)[2])
exp(confint.default(m1,"x1"))

#------------------------------------------------------------
#-- BINOMIAL GEE WITHOUT KC CORRECTION -----

m2<-geese(y~x1+x2,id=center,family=binomial(link="log"),
          corstr="exchangeable",data=df2)

# DF FOR T DISTRIBUTION TO CREATE 95% CI for RR
m2.cov.lz<-GEE.var.lz.BIN(y~x1+x2,id="center",data=df2)
df.lz.bin=2*(m2.cov.lz$cov.beta[2]^2)/m2.cov.lz$cov.var[5,5]
t.val.bin<-qt(.025,df=df.lz.bin,lower=F)

# RR and 95% t-approx CI
exp(m2$beta[2])
exp(m2$beta[2]+c(-1,1)*t.val.bin*sqrt(m2.cov.lz$cov.beta[2]))
```

```
#-- BINOMIAL GEE 95% CIs WITH KC CORRECTION -----
# DF FOR T DISTRIBUTION TO CREATE 95% CI
m2.cov.kc<-GEE.var.kc.BIN(y~x1+x2,id="center",data=df2)
df.kc.bin=2*(m2.cov.kc$cov.beta[2]^2)/m2.cov.kc$cov.var[5,5]
t.kc.bin<-qt(.025,df=df.kc.bin,lower=F)

# 95% t-approx CI WITH KC CORRECTION
exp(m2$beta[2]+c(-1,1)*t.kc.bin*sqrt(m2.cov.kc$cov.beta[2]))

#------------------------------------------------------------
#-- POISSON GEE WITHOUT KC CORRECTION -----
m3<-geese(y~x1+x2,id=center,family=poisson(link="log"),
          corstr="exchangeable",data=df2)

# DF FOR T DISTRIBUTION TO CREATE 95% CI for RR
m3.cov.lz<-GEE.var.lz(y~x1+x2,id="center",family=poisson,
                      corstr="exchangeable",data=df2)
df.lz.pois=2*(m3.cov.lz$cov.beta[2]^2)/m3.cov.lz$cov.var[5,5]
t.val.pois<-qt(.025,df=df.lz.pois,lower=F)

# RR and 95% t-approx CI
exp(m3$beta[2])
exp(m3$beta[2]+c(-1,1)*t.val.pois*sqrt(m3.cov.lz$cov.beta[2]))

#-- POISSON GEE 95% CIs WITH KC CORRECTION -----
# DF FOR T DISTRIBUTION TO CREATE 95% CI
m3.cov.kc<-GEE.var.kc(y~x1+x2,id="center",family=poisson,
                      corstr="exchangeable",data=df2)
df.kc.pois=2*(m3.cov.kc$cov.beta[2]^2)/m3.cov.kc$cov.var[5,5]
t.kc.pois<-qt(.025,df=df.kc.pois,lower=F)

# 95% t-approx CI WITH KC CORRECTION
exp(m3$beta[2]+c(-1,1)*t.kc.pois*sqrt(m3.cov.kc$cov.beta[2]))

#------------------------------------------------------------
#--- BINOMIAL GLMM WITH 95% WALD CI
m4<-glmer(y~x1+x2+(1|center),family=binomial(link="log"),nAGQ =10,data=df2)
exp(fixef(m4)[2])
exp(confint(m4,"x1",method="Wald"))

# GLMM BOOTSTRAP 95% CI FOR RR
m4.bs<-confint(m4,parm="x1",method="boot",type="parametric",use.u=F,
               boot.type="perc",nsim=3000)
exp(m4.bs)

#-------------------------------------------------------------------
#-- BAYESIAN BINOMIAL GLMM WITH MILDLY INFORMATIVE PRIORS -------------

# PREPARING DATA FOR STAN
dataIndexed = list(
  y =df2$y,
  nsub = length(df2$y),
  ncenter = length(unique(df2$center)),
  x1 = df2$x1,
  x2 = df2$x2,
  center = as.numeric(df2$center)
)
```

```
# STAN MODEL
bayescode<-"
#  HIERARCHICAL LOG BINOMIAL MODEL
## ASSUMES A MULTICENTER DESIGN
## ESTIMATES RELATIVE RISK OF TREATMENT/EXPOSURE
data {
int<lower=0> nsub;
int<lower=0> ncenter;
int y[nsub];
int center[nsub];
vector[nsub] x1; # treatment
vector[nsub] x2; # covariate
}

parameters {
real mu_b0;
vector[ncenter] b0;
real b1;
real b2;
real<lower=0> sigma;
}

transformed parameters{
vector<lower=0, upper=1>[nsub] p;
for(i in 1:nsub){
p[i] <- exp(b0[center[i]]+b1*x1[i]+b2*x2[i]);
}
}

model {
mu_b0 ~  normal(0.0, 10);
b0 ~  normal(mu_b0, sigma);
b1 ~  normal(0.0, 1);
b2 ~  normal(0.0, 1);
sigma ~   normal(0,1);

for(i in 1:nsub){
if (p[i] <1)
increment_log_prob(bernoulli_log(y[i],p[i]));
}
}
"
# CREATING INITIAL VALUES FOR MCMC CHAINS
mod1<-glm(y~x1+x2,family="poisson",data=df2)
betas<-coef(mod1)

inits <- function(betas.init,ncenter) {
  list(mu_b0=rnorm(1,mean=betas.init[1],sd=.5),
       b1=rnorm(1,mean=betas.init[2],sd=.5),
       b2=rnorm(1,mean=betas.init[3],sd=.5),
       b0=rep(-1.5,11),
       sigma=rtruncnorm(1,a=0,b=5))
}

n.center<-length(unique(df2$center))
n_chains=3
```

```
init_ll <- lapply(1:n_chains, function(id)
inits(betas.init=betas,ncenter=n.center))

# RUNNING MODEL
m6<-stan(model_code = bayescode, data=dataIndexed,
pars=c("mu_b0","b1","b2","sigma"),
         init = init_ll,chains=n_chains,iter=60000)

# CHECKING TRACE PLOTS OF PARAMETERS
traceplot(m6)

# POSTERIOR MEDIAN AND 95% INTERVAL FOR RR
m6.1<-extract(m6,pars='b1')$b1
quantile(exp(m6.1),c(.5,.025,.975))

#- END OF R CODE --------

## FUNCTION TO CALCULATE NEEDED INPUTS FOR DF FOR LOG BINOMIAL GEE ----------
# WITHOUT KC CORRECTION ----------------------
# NOTE THAT THE ARGUMENT "family = poisson" IS NOT AN ERROR BUT RATHER A
# TRICK FOR LOG LINK

GEE.var.lz.BIN<-function (formula, id = "subject", family = poisson, data,
                          corstr = "exchangeable")
{
  if (is.null(data$id)) {
    index <- which(names(data) == id)
    data$id <- data[, index]
  }
  init <- model.frame(formula, data)
  init$num <- 1:length(init[, 1])
  if (any(is.na(init))) {
    index <- na.omit(init)$num
    data <- data[index, ]
    m <- model.frame(formula, data)
    mt <- attr(m, "terms")
    data$response <- model.response(m, "numeric")
    mat <- as.data.frame(model.matrix(formula, m))
  }
  else {
    m <- model.frame(formula, data)
    mt <- attr(m, "terms")
    data$response <- model.response(m, "numeric")
    mat <- as.data.frame(model.matrix(formula, m))
  }
  gee.fit <- gee(formula, data = data, id = id, family =
binomial(link="log"),
                 corstr = corstr)
  beta_est <- gee.fit$coefficient
  alpha <- gee.fit$working.correlation[1, 2]
  len <- length(beta_est)
  len_vec <- len^2
  data$id <- gee.fit$id
  cluster <- cluster.size(data$id)
  ncluster <- max(cluster$n)
  size <- cluster$m
```

```
  mat$subj <- rep(unique(data$id), cluster$n)
  if (is.character(corstr)) {
    var <- switch(corstr, independence = cormax.ind(ncluster),
                  exchangeable = cormax.exch(ncluster, alpha), `AR-M` =
cormax.ar1(ncluster,

alpha), unstructured = summary(gee.fit)$working.correlation,
    )
  }
  else {
    print(corstr)
    stop("'working correlation structure' not recognized")
  }
  if (is.character(family)) {
    family <- switch(family, gaussian = "gaussian", binomial = "binomial",
                     poisson = "poisson")
  }
  else {
    if (is.function(family)) {
      family <- family()[[1]]
    }
    else {
      print(family)
      stop("'family' not recognized")
    }
  }
  cov.beta <- unstr <- matrix(0, nrow = len, ncol = len)
  step11 <- matrix(0, nrow = len, ncol = len)
  for (i in 1:size) {
    y <- as.matrix(data$response[data$id == unique(data$id)[i]])
    covariate <- as.matrix(subset(mat[, -length(mat[1, ])],
                             mat$subj == unique(data$id)[i]))
    var_i = var[1:cluster$n[i], 1:cluster$n[i]]
    if (family == "gaussian") {
      xx <- t(covariate) %*% solve(var_i) %*% covariate
      step11 <- step11 + xx
    }
    else if (family == "poisson") {
      D <- mat.prod(covariate, exp(covariate %*% beta_est))
      Vi <- diag(sqrt(c(exp(covariate %*% beta_est))),
                 cluster$n[i]) %*% var_i %*% diag(sqrt(c(exp(covariate %*%
                                                              beta_est))),
cluster$n[i])
      xx <- t(D) %*% solve(Vi) %*% D
      step11 <- step11 + xx
    }
    else if (family == "binomial") {
      D <- mat.prod(covariate, exp(covariate %*% beta_est)/((1 +
                                                              exp(covariate
%*% beta_est))^2))
      Vi <- diag(sqrt(c(exp(covariate %*% beta_est)/(1 +
                                                      exp(covariate %*%
beta_est))^2)), cluster$n[i]) %*%
        var_i %*% diag(sqrt(c(exp(covariate %*% beta_est)/(1 +
                                                            exp(covariate
%*% beta_est))^2)), cluster$n[i])
      xx <- t(D) %*% solve(Vi) %*% D
```

```
      step11 <- step11 + xx
    }
  }
  step12 <- matrix(0, nrow = len, ncol = len)
  step13 <- matrix(0, nrow = len_vec, ncol = 1)
  step14 <- matrix(0, nrow = len_vec, ncol = len_vec)
  p <- matrix(0, nrow = len_vec, ncol = size)
  for (i in 1:size) {
    y <- as.matrix(data$response[data$id == unique(data$id)[i]])
    covariate <- as.matrix(subset(mat[, -length(mat[1, ])],
                              mat$subj == unique(data$id)[i]))
    var_i = var[1:cluster$n[i], 1:cluster$n[i]]
    if (family == "gaussian") {
      xy <- t(covariate) %*% solve(var_i) %*% (y - covariate %*%
                                               beta_est)
      step12 <- step12 + xy %*% t(xy)
      step13 <- step13 + vec(xy %*% t(xy))
      p[, i] <- vec(xy %*% t(xy))
    }
    else if (family == "poisson") {
      D <- mat.prod(covariate, exp(covariate %*% beta_est))
      Vi <- diag(sqrt(c(exp(covariate %*% beta_est))),
                 cluster$n[i]) %*% var_i %*% diag(sqrt(c(exp(covariate %*%
                                                            beta_est))),
cluster$n[i])
      xy <- t(D) %*% solve(Vi) %*% (y - exp(covariate %*%
                                             beta_est))
      step12 <- step12 + xy %*% t(xy)
      step13 <- step13 + vec(xy %*% t(xy))
      p[, i] <- vec(xy %*% t(xy))
    }
    else if (family == "binomial") {
      D <- mat.prod(covariate, exp(covariate %*% beta_est)/((1 +
                                                            exp(covariate
%*% beta_est))^2))
      Vi <- diag(sqrt(c(exp(covariate %*% beta_est)/(1 +
                                                      exp(covariate %*%
beta_est))^2)), cluster$n[i]) %*%
        var_i %*% diag(sqrt(c(exp(covariate %*% beta_est)/(1 +
                                                          exp(covariate
%*% beta_est))^2)), cluster$n[i])
      xy <- t(D) %*% solve(Vi) %*% (y - exp(covariate %*%
                                             beta_est)/(1 + exp(covariate
%*% beta_est)))
      step12 <- step12 + xy %*% t(xy)
      step13 <- step13 + vec(xy %*% t(xy))
      p[, i] <- vec(xy %*% t(xy))
    }
  }
  for (i in 1:size) {
    dif <- (p[, i] - step13/size) %*% t(p[, i] - step13/size)
    step14 <- step14 + dif
  }
  cov.beta <- solve(step11) %*% (step12) %*% solve(step11)
  cov.var <- size/(size - 1) * kronecker(solve(step11), solve(step11)) %*%
    step14 %*% kronecker(solve(step11), solve(step11))
  return(list(cov.beta = diag(cov.beta), cov.var = cov.var))
```

```
}

## FUNCTION TO CALCULATE NEEDED INPUTS FOR DF FOR LOG BINOMIAL GEE ----------
# WITH KC CORRECTION -----------------------
# NOTE THAT THE ARGUMENT "family = poisson" IS NOT AN ERROR BUT RATHER A
# TRICK FOR LOG LINK

GEE.var.kc.BIN<-function (formula, id, family = poisson, data, corstr =
"exchangeable")
{
  if (is.null(data$id)) {
    index <- which(names(data) == id)
    data$id <- data[, index]
  }
  init <- model.frame(formula, data)
  init$num <- 1:length(init[, 1])
  if (any(is.na(init))) {
    index <- na.omit(init)$num
    data <- data[index, ]
    m <- model.frame(formula, data)
    mt <- attr(m, "terms")
    data$response <- model.response(m, "numeric")
    mat <- as.data.frame(model.matrix(formula, m))
  }
  else {
    m <- model.frame(formula, data)
    mt <- attr(m, "terms")
    data$response <- model.response(m, "numeric")
    mat <- as.data.frame(model.matrix(formula, m))
  }
  gee.fit <- gee(formula, data = data, id = id, family =
binomial(link="log"),
                 corstr = corstr)
  beta_est <- gee.fit$coefficient
  alpha <- gee.fit$working.correlation[1, 2]
  len <- length(beta_est)
  len_vec <- len^2
  data$id <- gee.fit$id
  cluster <- cluster.size(data$id)
  ncluster <- max(cluster$n)
  size <- cluster$m
  mat$subj <- rep(unique(data$id), cluster$n)
  if (is.character(corstr)) {
    var <- switch(corstr, independence = cormax.ind(ncluster),
                  exchangeable = cormax.exch(ncluster, alpha), `AR-M` =
cormax.ar1(ncluster,

alpha), unstructured = summary(gee.fit)$working.correlation,
    )
  }
  else {
    print(corstr)
    stop("'working correlation structure' not recognized")
  }
  if (is.character(family)) {
    family <- switch(family, gaussian = "gaussian", binomial = "binomial",
                     poisson = "poisson")
```

```
  }
  else {
    if (is.function(family)) {
      family <- family()[[1]]
    }
    else {
      print(family)
      stop("'family' not recognized")
    }
  }
  cov.beta <- unstr <- matrix(0, nrow = len, ncol = len)
  step11 <- matrix(0, nrow = len, ncol = len)
  for (i in 1:size) {
    y <- as.matrix(data$response[data$id == unique(data$id)[i]])
    covariate <- as.matrix(subset(mat[, -length(mat[1, ])],
                                  mat$subj == unique(data$id)[i]))
    var_i = var[1:cluster$n[i], 1:cluster$n[i]]
    if (family == "gaussian") {
      xx <- t(covariate) %*% solve(var_i) %*% covariate
      step11 <- step11 + xx
    }
    else if (family == "poisson") {
      D <- mat.prod(covariate, exp(covariate %*% beta_est))
      Vi <- diag(sqrt(c(exp(covariate %*% beta_est))),
                 cluster$n[i]) %*% var_i %*% diag(sqrt(c(exp(covariate %*%
                                                              beta_est))),
cluster$n[i])
      xx <- t(D) %*% solve(Vi) %*% D
      step11 <- step11 + xx
    }
    else if (family == "binomial") {
      D <- mat.prod(covariate, exp(covariate %*% beta_est)/((1 +
                                                              exp(covariate
%*% beta_est))^2))
      Vi <- diag(sqrt(c(exp(covariate %*% beta_est)/(1 +
                                                      exp(covariate %*%
beta_est))^2)), cluster$n[i]) %*%
        var_i %*% diag(sqrt(c(exp(covariate %*% beta_est)/(1 +
                                                            exp(covariate
%*% beta_est))^2)), cluster$n[i])
      xx <- t(D) %*% solve(Vi) %*% D
      step11 <- step11 + xx
    }
  }
  step12 <- matrix(0, nrow = len, ncol = len)
  step13 <- matrix(0, nrow = len_vec, ncol = 1)
  step14 <- matrix(0, nrow = len_vec, ncol = len_vec)
  p <- matrix(0, nrow = len_vec, ncol = size)
  for (i in 1:size) {
    y <- as.matrix(data$response[data$id == unique(data$id)[i]])
    covariate <- as.matrix(subset(mat[, -length(mat[1, ])],
                                  mat$subj == unique(data$id)[i]))
    var_i = var[1:cluster$n[i], 1:cluster$n[i]]
    if (family == "gaussian") {
      xy <- t(covariate) %*% solve(var_i) %*%
mat.sqrt.inv(cormax.ind(cluster$n[i]) -
```

```
                                                              covariate %*%
solve(step11) %*% t(covariate) %*%
                                                              solve(var_i))
%*% (y - covariate %*% beta_est)
      step12 <- step12 + xy %*% t(xy)
      step13 <- step13 + vec(xy %*% t(xy))
      p[, i] <- vec(xy %*% t(xy))
    }
    else if (family == "poisson") {
      D <- mat.prod(covariate, exp(covariate %*% beta_est))
      Vi <- diag(sqrt(c(exp(covariate %*% beta_est))),
                 cluster$n[i]) %*% var_i %*% diag(sqrt(c(exp(covariate %*%
                                                                beta_est))),
cluster$n[i])
      xy <- t(D) %*% solve(Vi) %*% mat.sqrt.inv(cormax.ind(cluster$n[i]) -
                                                  D %*% solve(step11) %*%
t(D) %*% solve(Vi)) %*%
        (y - exp(covariate %*% beta_est))
      step12 <- step12 + xy %*% t(xy)
      step13 <- step13 + vec(xy %*% t(xy))
      p[, i] <- vec(xy %*% t(xy))
    }
    else if (family == "binomial") {
      D <- mat.prod(covariate, exp(covariate %*% beta_est)/((1 +
                                                                exp(covariate
%*% beta_est))^2))
      Vi <- diag(sqrt(c(exp(covariate %*% beta_est)/(1 +
                                                          exp(covariate %*%
beta_est))^2)), cluster$n[i]) %*%
        var_i %*% diag(sqrt(c(exp(covariate %*% beta_est)/(1 +
                                                              exp(covariate
%*% beta_est))^2)), cluster$n[i])
      xy <- t(D) %*% solve(Vi) %*% mat.sqrt.inv(cormax.ind(cluster$n[i]) -
                                                  D %*% solve(step11) %*%
t(D) %*% solve(Vi)) %*%
        (y - exp(covariate %*% beta_est)/(1 + exp(covariate %*%
                                                    beta_est)))
      step12 <- step12 + xy %*% t(xy)
      step13 <- step13 + vec(xy %*% t(xy))
      p[, i] <- vec(xy %*% t(xy))
    }
  }
  for (i in 1:size) {
    dif <- (p[, i] - step13/size) %*% t(p[, i] - step13/size)
    step14 <- step14 + dif
  }
  cov.beta <- solve(step11) %*% (step12) %*% solve(step11)
  cov.var <- size/(size - 1) * kronecker(solve(step11), solve(step11)) %*%
    step14 %*% kronecker(solve(step11), solve(step11))
  return(list(cov.beta = diag(cov.beta), cov.var = cov.var))
}
```

OpenBUGS Code

```
model {
for(i in 1:nsubject) {
y[i] ~ dbern(p[i])
log(p[i]) <- b0[center[i]] + b1*x1[i]+b2*x2[i]
}
RR <- exp(b1)

for(j in 1:ncenter){
b0[j] ~  dnorm(mu_b0, tau)
}
mu_b0 ~  dnorm(0, .01)
b1 ~  dnorm(0, 1)
b2 ~  dnorm(0, 1)
sigma ~   dnorm(0,1)I(0,)
tau<- 1/(sigma*sigma)

for (i in 1:nsubject){
ones[i] <- 1
ones[i] ~ dbern(C1[i])
C1[i] <- step(1-p[i])
}
}
```
